# Beliefs About Medication and Uptake of Preventive Therapy in Women at Increased Risk of Breast Cancer: Results From a Multicenter Prospective Study

**DOI:** 10.1016/j.clbc.2018.10.008

**Published:** 2019-02

**Authors:** Rachael Jane Thorneloe, Rob Horne, Lucy Side, Michael Scott Wolf, Samuel George Smith, ENGAGE Investigators, Vanessa Adamson, Sarah Ainsworth, Malin Akerlund, Ivanna Baker, Julian Barwell, Jayne Beesley, Lisa Brock, Chrissie Butcher, Janice Carpenter, Martyn Clark, Shirley Cocks, Veronica Conteh, Martina Coulding, Sue Darby, Angela Duckworth, Gareth Evans, Catherine Fensom, Julie Fletcher, Kate Foster, Sara Grieg, Elaine Gullaksen, Jana Gurasashvili, Lisa Hardstaff, Rachel Hart, Kathryn Hoare, Jonathan Hoffman, Christopher Holcombe, Lynne Horton, Antony Howell, Farah Islam, Emma Jenkinson, Karen Jewers, Manisha Joshi, Amy Kirkby, Peter Kneeshaw, Natalie Knife, Jalal Kokan, Jin Li, Nicola Lunt, Douglas Macmillan, Karen Makinson, Evangelos Mallidis, Sarah Manyangadze, Charity Masvaure, Raksha Mistry, Alice Ngumo, Jane Ooi, Ashraf Patel, Vanessa Pope, Laura Price, Fiona Rabson, Lisa Richardson, Stephanie Ridgway, Karen Riley, Lorraine Roberts, Janet Ryan-Smith, Vian Salih, Nicky Scott, Mike Shere, Andrew Sloan, Nita Solanky, Amanda Taylor, Dinesh Thekkinkattil, Heather Thomas, Mangesh Thorat, Barbara Townley, Jayant S. Vaidya, Lynda Wagstaff, Shane Walsh, Lynsey Waring, Donna Watterson, Charlotte Westley, Lesley Wilkinson, Nicola Willis, Julia Wiseman

**Affiliations:** 1 Leeds Institute of Health Sciences, University of Leeds, Leeds, United Kingdom; 2 Centre for Behavioural Medicine, School of Pharmacy, University College London, London, United Kingdom; 3 Wessex Clinical Genetics Service, University Hospitals Southampton, Southampton, United Kingdom; 4 Division of General Internal Medicine and Geriatrics, Northwestern University, Evanston, IL

**Keywords:** BMQ, Breast cancer prevention, Chemoprevention, Decision-making, Medication beliefs

## Abstract

**Introduction:**

Uptake of preventive therapies for breast cancer is low. We examined whether women at increased risk of breast cancer can be categorized into groups with similar medication beliefs, and whether belief group membership was prospectively associated with uptake of preventive therapy.

**Patients and Methods:**

Women (n = 732) attending an appointment to discuss breast cancer risk were approached; 408 (55.7%) completed the Beliefs About Medicines and the Perceived Sensitivity to Medicines questionnaires. Uptake of tamoxifen at 3 months was reported in 258 (63.2%). The optimal number of belief groups were identified using latent profile analysis.

**Results:**

Uptake of tamoxifen was 14.7% (38/258). One in 5 women (19.4%; 78/402) reported a strong need for tamoxifen. The model fit statistics supported a 2-group model. Both groups held weak beliefs about their need for tamoxifen for current and future health. Group 2 (38%; 154/406 of the sample) reported stronger concerns about tamoxifen and medicines in general, and stronger perceived sensitivity to the negative effects of medicines compared with group 1 (62%; 252/406). Women with low necessity and lower concerns (group 1) were more likely to initiate tamoxifen (18.3%; 33/180) than those with low necessity and higher concerns (group 2) (6.4%; 5/78). After adjusting for demographic and clinical factors, the odds ratio was 3.37 (95% confidence interval, 1.08-10.51; *P* = .036).

**Conclusion:**

Uptake of breast cancer preventive therapy was low. A subgroup of women reported low need for preventive therapy and strong medication concerns. These women were less likely to initiate tamoxifen. Medication beliefs are targets for supporting informed decision-making.

## Micro-Abstract

Preventive therapies, such as tamoxifen, are a risk reduction option for women at increased risk of breast cancer. Little is known about the psychological factors influencing the decision to use chemoprevention. Using latent profile analysis, women who reported a low need for preventive therapy and strong medication concerns were less likely to initiate tamoxifen treatment. Medication beliefs are targets for supporting informed decision-making.

## Introduction

Breast cancer is the most common cancer in women worldwide.^1^ Preventive therapy is a risk reduction approach for women at increased risk of breast cancer. In a meta-analysis of 9 randomized trials, women at increased risk of breast cancer had at least a 30% lower risk of the disease if they used selective estrogen receptor modulators.^2^ The IBIS-I (International Breast Cancer Intervention Study) indicated the preventive effect of tamoxifen lasts for at least 20 years.^3^ The effectiveness of preventive therapy depends on adequate uptake but initiation rates remain low.^4^, ^5^, ^6^, ^7^

Individual’s beliefs about medication are modifiable drivers of treatment decision-making.^8^ These beliefs include perceptions of personal need for medication (necessity beliefs) and concerns about its usage (concern beliefs), as well as more general concerns relating to the nature of medications and how they are used by doctors. The Beliefs About Medicines Questionnaire (BMQ) is the tool most commonly used to assess and quantify medication beliefs.^8^, ^9^ Women’s concerns about side effects are a barrier to initiating preventive therapy.^5^, ^10^ However, there can be heterogeneity in individual’s beliefs.^11^ Understanding subgroup differences in medication beliefs can support the development of personalized interventions.

The objectives of this study were to: (1) assess whether women at increased risk of breast cancer can be categorized into groups with similar medication beliefs; (2) determine whether sociodemographic and clinical variables are related to medication belief group membership and; (3) examine whether medication belief groups are associated with tamoxifen uptake.

## Patients and Methods

### Patients

Women were approached after their appointment at 1 of the following clinic types; family history clinic (n = 12), breast clinic (n = 4), clinical genetic centers (n = 3), and a family history clinic with genetics support (n = 1). In the United Kingdom, women are referred to secondary care if their general practitioner (family doctor) believes they are likely to meet National Institute for Health and Care Excellence (NICE) criteria for breast cancer risk.^12^ Recruitment took place at 20 clinics in England between September 2015 and December 2016. Eligibility criteria included: women aged 18 years or older; English-speaking; had discussed preventive therapy with a health care professional; were classified as having a moderately high or high risk of breast cancer according to NICE guidelines^12^; and had no known contraindications for tamoxifen use. Women were excluded if they were unable to consent, read English, or had a previous diagnosis of breast cancer.

## Materials

Women were invited to complete a baseline survey containing the following measures: the BMQ^9^ is used to assess perceptions about personal need for tamoxifen (3 items, specific necessity); concerns about negative effects from tamoxifen (6 items, specific concerns); beliefs relating to the nature of medication (4 items, general harmfulness); and beliefs about how they are used by doctors (4 items, general overuse). The BMQ was adapted for use in chemoprevention decision-making. Each item is scored on a 5-point scale (“strongly disagree: [= 1] to “strongly agree” [= 5]), with higher scores indicating stronger medication beliefs. A mean score was calculated for each subscale, with scores ranging from 1 to 5. The proportion of women who agreed (= 4) or strongly agreed (= 5) with each item within the subscales were also examined.

The Perceived Sensitivity to Medicines (PSM) scale^13^ is used to assesses perceived sensitivity to potential adverse effects of medicines (5 items). Each item is scored on a 5-point scale (“strongly disagree” [=1] to “strongly agree” [= 5]), with higher scores indicating higher perceived sensitivity to the negative effects of medicines. A mean score was calculated, with scores ranging from 1 to 5. The proportion of women who agreed (= 4) or strongly agreed (= 5) with each individual scale item was examined.

The baseline survey obtained the following data: marital status; ethnicity; education level; employment status; nulliparity; and self-reported health. Age was calculated from date of birth provided from National Health Service records; women were coded as ≤35 years; 36 to 49 years and; ≥50 years for analysis. Index of Multiple Deprivation scores were calculated from participant postcodes, and women were classified into tertiles of neighborhood deprivation.^14^ Breast cancer risk category (moderately high or high) as outlined in the NICE guidelines, was provided by clinic staff (with participant consent).^12^ Uptake of tamoxifen was assessed in the 3-month follow-up questionnaire. Women were classified as initiating tamoxifen if they reported having a prescription for tamoxifen from their general practitioner or were currently taking tamoxifen. This is because some women might not have had the opportunity to collect their prescription and start treatment at the time of the 3-month follow-up period.

### Analysis

The analysis was preregistered.^15^ The association between the BMQ subscales and the PSM scale were analyzed using Pearson correlation coefficients. Differences in medication beliefs between those who completed the baseline survey and women who returned a baseline and follow-up survey were analyzed using *t* tests. Theory-driven latent profile analysis (LPA) was used to investigate whether women could be categorized into medication belief groups. LPA is used to categorize individuals with similar profiles on a set of continuous variables (BMQ and PSM scales) into discrete groups represented by a categorical latent variable (medication belief groups). Participants’ mean scores for the BMQ subscales and the PSM scale were included in the LPA analysis. Two participants had missing data for all 5 variables and were excluded from analysis (n = 406 included in baseline analysis). Model fit statistics for LPA models with 1 through 5 class solutions were examined. These were the Akaike Information Criterion (AIC) and the Bayesian Information Criterion (BIC), where smaller values indicate a better fit. The Vuong–Lo–Mendell–Rubin likelihood ratio test and the Lo–Mendell–Rubin adjusted likelihood ratio test were used to compare the current model with a model with 1 less latent class. Entropy provides a measure of the classification quality of the model, with values approaching 1 indicating a good separation of classes.

Two planned sensitivity analyses were performed. The LPA model was run with and without the PSM scale. The LPA model was also run on individuals who provided baseline and 3-month follow-up data on tamoxifen uptake (n = 258) to ensure that the reduction in sample size would not bias the results.

A multivariable logistic regression model was used to examine the association between participant characteristics and medication belief group membership. Multivariable logistic regression was also used to examine the role of medication belief group membership on uptake. The analysis was done using Mplus 7^16^ and SPSS version 24.0 (IBM Corp). Statistical significance was set at a 2-sided *P* < .05.

### Ethical Approval

Ethical approval was awarded by the National Research Ethics Service Committee North West–Preston (14/NW/1408). Informed consent was implied with the return of a questionnaire.

### Availability of Data and Material

Participants did not provide explicit consent for their data to be shared in public repositories. Therefore, data may not be made publicly available because of ethical restrictions. We can share the anonymized version of the data with individual qualified researchers upon request. Data requests may be sent to the corresponding author of this report.

## Results

**Supplemental Figure 1:**
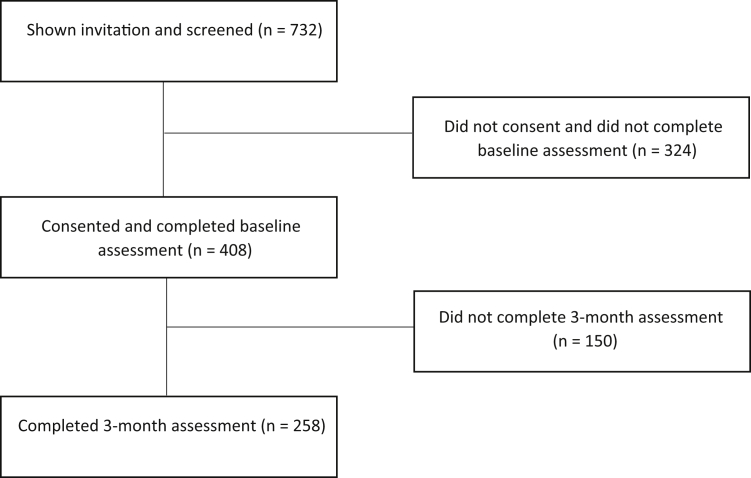
Recruitment Flow Diagram

In total, 732 women were invited to complete a survey; 408 women (55.7%) returned the baseline survey (Table 1) and 258 (63.2%) women provided uptake data at least 3 months after their appointment (see Supplemental Figure 1 in the online version). Demographic and clinical differences between responders and nonresponders and between those who did and did not provide 3-month data are published elsewhere.^6^ There were no differences between responders and nonresponders with regard to clinical risk, socioeconomic status (SES), or age group. Women were more likely to provide follow-up data if they were from a higher SES group. There were no differences in medication beliefs between women who provided baseline data and those who provided baseline and 3-month data (see Supplemental Table 1 in the online version).

**Table 1:** Demographic, Clinical, and Psychological Variables at Baseline (n = 408)

Variable	Value
**Demographic and Clinical**	
Age	45.30 (±7.82)
Children	
Yes	314 (77.0)
No	94 (23.0)
Ethnic group	
White	384 (95.5)
Other	18 (4.5)
Education level	
Degree or above	176 (44.2)
Below degree level	222 (55.8)
Health status	
Poor	16 (4.0)
Fair	78 (19.5)
Good	240 (60.0)
Excellent	66 (16.5)
Risk level	
Moderate	243 (59.6)
High	159 (39.0)
Unclear	6 (1.4)
SES	
Low (most deprived)	120 (29.9)
Middle	131 (32.7)
High (least deprived)	150 (37.4)
Employment	
Full-time	348 (85.3)
All other employment	60 (14.7)
Marital status	
Married or cohabiting	298 (74.3)
Unmarried	103 (25.7)
**Beliefs about Medicines Questionnaire**	
Specific necessity	2.63 (±0.77)
Specific concerns	3.11 (±0.60)
General overuse	2.68 (±0.73)
General harmfulness	2.28 (±0.61)
**Perceived Sensitivity to Medicines**	
Score	2.34 (±0.77)

Data are presented as mean (±SD) for continuous variables and n (%) for categorical variables.

Abbreviation: SES = socioeconomic status.

### Beliefs About Medication and Perceived Sensitivity to its Effects

Women reported low perceived need for tamoxifen; 19.4% (78/402) believed their current health depends on them taking tamoxifen and 18.2% (73/401) believed they would become very ill without it (Table 2). Concerns about tamoxifen were common; 72.4% (291/402) worried about its long-term effects and 56.9% (230/404) believed tamoxifen use would result in unpleasant side effects. A significant proportion of women reported poor understanding about tamoxifen; 22.6% (91/402) believed tamoxifen was a “mystery” to them. Perceptions of perceived need for tamoxifen were unrelated to concerns about its usage (see Supplemental Table 2 in the online version).

**Table 2:** Beliefs About Medication and Perceived Sensitivity to its Effects for the Entire Sample and Medication Belief Groups (n = 408)

	Sample (n = 408)	Group 1 (Low Need, Lower Concerns) (62%; n = 252)	Group 2 (Low Need, Higher Concerns) (38%; n = 154)
**BMQ Specific Necessity Beliefs**			
1. My current health depends on me taking tamoxifen	19.4	21.4	16.2
2. Without tamoxifen, I could become very ill	18.2	18.1	18.4
3. My future health depends on me taking tamoxifen	22.1	25.3	16.9
**BMQ Specific Concern Beliefs**			
1. Taking tamoxifen would worry me	61.3	56.9	68.6
2. I worry about the long-term effects of tamoxifen	72.4	66.3	82.4
3. Tamoxifen is a mystery to me	22.6	17.7	30.5
4. Taking tamoxifen would disrupt my life	23.8	21.6	27.3
5. I worry I would become dependent on tamoxifen	9.2	6.0	14.5
6. Tamoxifen would give me unpleasant side effects	56.9	52.0	64.9
**BMQ General Overuse Beliefs**			
1. Doctors use too many medicines	28.9	10.4	59.1
2. Natural remedies are safer than medicines	17.0	6.0	35.1
3. Doctors place too much trust in medicines	14.3	2.0	34.4
4. If doctors had more time with patients they would prescribe fewer medicines	35.3	16.7	66.0
**BMQ General Harmfulness Beliefs**			
1. People who take medicines should stop for a while every now and again	23.7	10.8	44.8
2. Most medicines are addictive	13.3	3.2	29.9
3. Medicines do more harm than good	3.2	0.4	7.9
4. All medicines are poisons	5.9	1.2	13.6
**PSM**			
1. My body is very sensitive to medicines	22.8	17.1	32.0
2. My body over-reacts to medicines	8.9	5.2	14.9
3. I usually have stronger reactions to medicines than most people	7.2	4.8	11.0
4. I have had a bad reaction to medicines in the past	24.2	21.0	29.4
5. Even very small amounts of medicines can upset my body	10.7	8.0	15.0

Data are the percentage who agreed or strongly agreed with each statement; reference category: strongly disagree, disagree, and unsure.

Abbreviations: BMQ = Beliefs about Medicines Questionnaire; PSM = Perceived Sensitivity to Medicines Scale.

A significant proportion of women reported concerns about the nature of medicines and how they are used by doctors. This included the belief that doctors use too many medicines (28.9%; 117/405) and would prescribe fewer medicines if they had more time with patients (35.3%; 143/405). Some women also reported heightened sensitivity to the effects of medication; 22.8% (92/404) reported that they were particularly sensitive to medicines and they have had reactions to medicines in the past, with 10.7% (43/403) believing that even very small amounts of medication can upset their body.

### Medication Belief Groups

Model fit statistics for 1 through 5 class solutions are presented in Supplemental Table 3 in the online version. Although the AIC, BIC, and entropy values supported a 3-class solution, the log ratio (LR) tests were nonsignificant, suggesting that extraction of 3 classes did not improve model fit above a 2-class solution. Furthermore, the second class was a small group (n = 14). A 2-class solution was selected; both LR tests were significant with good sample sizes within each latent class. Excluding the PSM scale did not improve model fit (see Supplemental Table 4 in the online version). Rerunning the analysis using only participants who had completed baseline and had 3-month uptake data indicated a 2-class solution with similar medication belief profiles (see Supplemental Table 5 in the online version).

**Figure 1:**
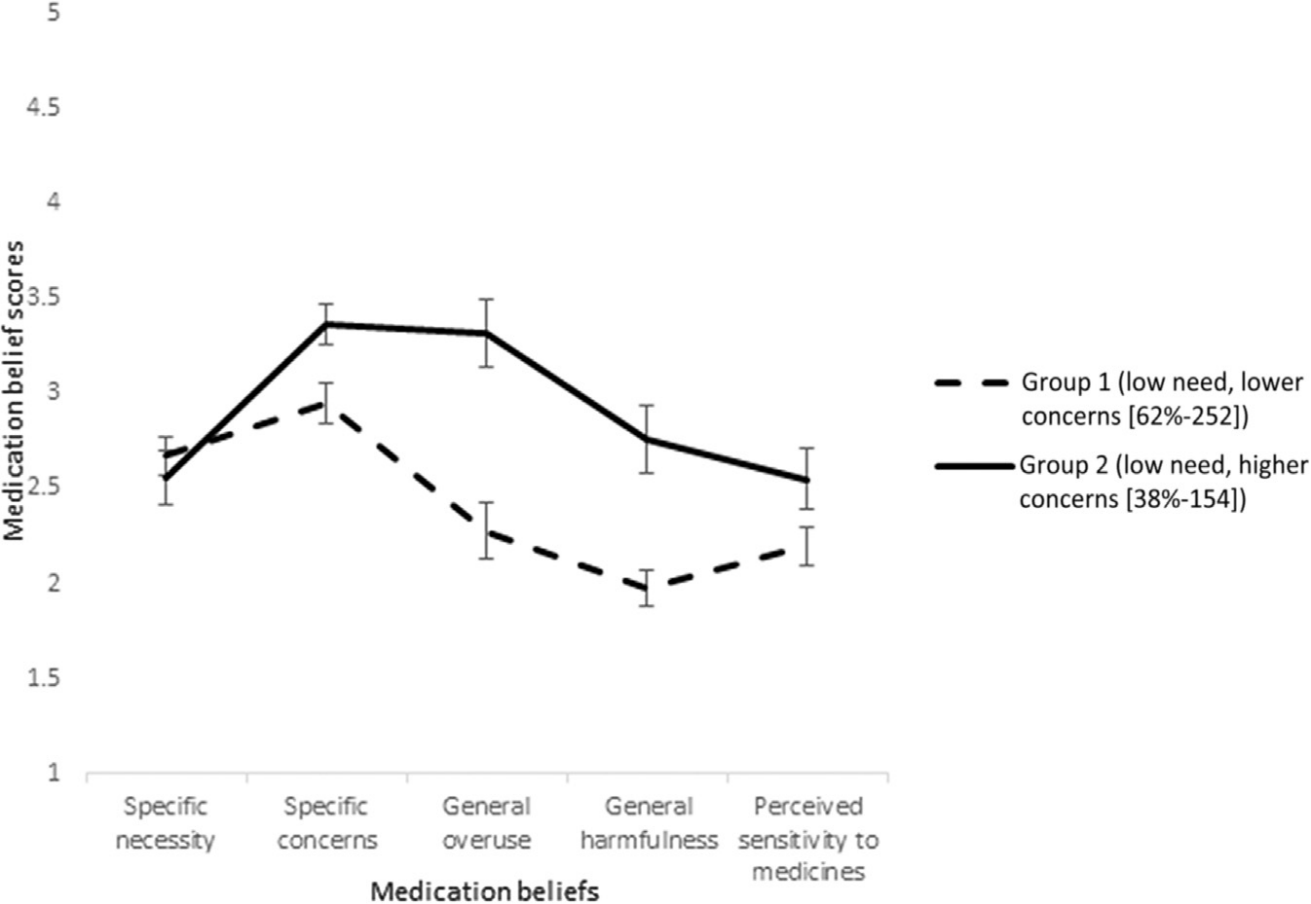
Sample Means [95% CI] of Medication Beliefs for the 2-Class Solution (n = 406). Chart Shows Differences in Medication Beliefs Between Group 1 (Low Need, Lower Concerns) and Group 2 (Low Need, Higher Concerns)

Sample means (95% confidence interval [CI]) of medication beliefs for the 2-class solution are presented in Figure 1. Both medication belief groups perceived a low need for tamoxifen (subscale: specific necessity), but differed in their medication concerns and perceived sensitivity to medicines. Women classified into group 2 (38%; 154) reported the strongest concerns about tamoxifen and medicines in general, as well as stronger perceived sensitivity to the effects of medicines, compared with women classified into group 1 (62%; 252). The largest difference between the groups was for concerns about the overuse and harmfulness of medicines in general (Table 2). A higher proportion of women classified into group 2 (low necessity and higher concerns) believed that doctors use too many (59.1% [91/154] vs. 10.4% [26/251]) and place too much trust in medicines (34.4% [53/154] vs. 2.0% [5/251]), and would prescribe fewer medicines if they had more time with patients (66% [101/153] vs. 16.7% [42/252]). A higher proportion of women in group 2 also believed that medicines are poisons (13.6% [21/154] vs. 1.2% [3/252]), addictive (29.9% [46/154] vs. 3.2% [8/252]), and people who take medicines should stop for a while every now and again (44.8% [69/154] vs. 10.8% [27/251]).

### Factors Related to Medication Belief Group Membership

Women classified into group 2 (low need, higher concerns) were more likely to be: aged 50 years or older (vs. 36-49 years), from nonwhite ethnic groups (vs. white ethnic group), not working full-time (vs. full-time employment), and unmarried (vs. married or cohabiting; see Supplemental Table 6 in the online version). Only age (50 years or older vs. 36-49 years) remained significantly associated with medication belief group membership in multivariable analyses (odds ratio [OR], 0.56; 95% CI, 0.34-0.93; *P* = .024).

### Predictors of Tamoxifen Uptake

Uptake of chemoprevention was 14.7% (38/258); 31 women were currently taking tamoxifen and 7 women reported having a prescription. Uptake according to clinic setting is presented in Supplemental Table 7 in the online version. Women classified into group 1 (low necessity, lower concerns) were more likely to initiate tamoxifen (18.3%; 33/180) than those classified into group 2 (low necessity, higher concerns) (6.4%; 5/78). After adjusting for demographic and clinical factors, the OR was 3.37 (95% CI, 1.08-10.51; *P* = .036; Table 3).

**Table 3:** Uptake of Tamoxifen According to Participant Characteristics and Univariable and Multivariable Logistic Regression Model (n = 258)

	Uptake, n (%)	Univariable		Multivariable	
		OR (95% CI)	*P*	OR (95% CI)	*P*
**Age**					
≤35 Years^a^	1 (3.8)^a^	–		–	
36-49 Years	29 (17.3)	1.46 (0.63-3.39)	.378	1.19 (0.44-3.18)	.731
≥50 Years	8 (12.5)	Ref		Ref	
**Children**					
Yes	36 (17.6)	5.43 (1.26-23.34)	**.023**	3.66 (0.76-17.64)	.106
No	2 (3.8)	Ref		Ref	
**Ethnic Group**^a^					
White	37 (15)	–	–	–	–
Other	1 (11.1)	–	–	–	–
**Education Level**					
Degree or above	20 (17.2)	1.41 (0.71-2.82)	.327	1.50 (0.66-3.42)	.335
Below degree level	18 (12.9)	Ref		Ref	
**Health Status**					
Poor^a^	0	–		–	
Fair	5 (10.6)	0.68 (0.20-2.32)	.538	0.53 (0.13-2.13)	.372
Good	25 (16.6)	1.13 (0.46-2.82)	.787	0.97 (0.37-2.60)	.958
Excellent	7 (14.9)	Ref		Ref	
**Risk Level**					
Moderate	24 (15.1)	1.05 (0.52-2.15)	.885	0.84 (0.38-1.82)	.651
High	14 (14.4)	Ref		Ref	
Unclear^a^	0	–			
**SES**					
Low (most deprived)	7 (11.9)	0.78 (0.30-2.03)	.613	1.23 (0.44-3.39)	.695
Middle	14 (16.3)	1.13 (0.52-2.47)	.759	1.38(0.57-3.33)	.479
High (least deprived)	16 (14.7)	Ref		Ref	
**Employment**					
Full-time	32 (14.5)	Ref		Ref	
All other employment	6 (16.2)	1.14 (0.44-2.96)	.783	1.82 (0.63-5.22)	.269
**Marital Status**					
Married or cohabiting	33 (16.7)	2.16 (0.80-5.81)	.127	1.47 (0.44-4.93)	.534
Unmarried	5 (8.5)	Ref		Ref	
**Medication Belief Group**					
Group 1 (low need, lower concerns)	33 (18.3)	3.28 (1.23-8.75)	**.018**	3.37 (1.08-10.51)	**.036**
Group 2 (low need, higher concerns)	5 (6.4)	Ref		Ref	

Bold *P* values indicate statistical significance *P* < .05.

Abbreviations: OR = odds ratio; Ref = reference; SES = socioeconomic status.

a Category not included in univariable and multivariable analyses because of insufficient cases; the multivariable model included 213 respondents.

## Discussion

In this United Kingdom multicenter study, only 1 in 5 women at increased risk of breast cancer reported a strong need for tamoxifen preventive therapy. More than 70% of women reported strong worries about its long-term effects and more than half reported concerns about potential unpleasant side effects. A subgroup of women, accounting for almost two-fifths of the sample, reported the strongest medication concerns and perceived sensitivity to medicines. Women with low necessity and lower concerns were more likely to initiate tamoxifen than those with low necessity and higher concerns.

It is important to determine whether preventive therapies can create or exacerbate existing inequalities in breast cancer outcomes.^17^ We have previously shown within this cohort that there are no sociodemographic differences in tamoxifen uptake.^6^ In this study, medication belief group membership was associated with key indicators of SES, which might help identify those who would most benefit from additional decision-making support.

Medication beliefs are key modifiable determinants of treatment decision-making.^8^ Beliefs about breast cancer risk and its treatment are complex and influenced by family experiences of cancer and medication use.^6^ We have illustrated the specific medication beliefs held by individuals at increased risk, with the identification of subgroup differences having implications for supporting informed decision-making. Perceived need for tamoxifen was low, suggesting intervention strategies should focus on communicating the role tamoxifen could play in cancer prevention, while balancing this with information about harms and respecting women’s decision to decline. Although women who reported low need and lower concerns (group 1) were more likely to initiate tamoxifen, uptake was still low in this group. For those who initiate tamoxifen, continued uncertainty about personal need might result in lower adherence, which has been shown to be problematic in clinical trials.^18^, ^19^, ^20^

An important subgroup of women reported low need for tamoxifen and stronger medication concerns, and these beliefs influenced uptake decisions. This group might benefit from additional support that focuses on eliciting and addressing unresolved medication concerns.^21^ Treatment expectations have been shown to increase the risk of treatment-specific side effects and nonadherence in the context of secondary breast cancer prevention.^22^ Our study shows how previous treatment expectations can influence primary prevention decision-making and emphasizes the need for clinicians to address concerns and ensure realistic treatment expectations.

### Strengths and Limitations

The participation of more than 400 women from 20 centers across England reflects the experiences of treatment decision-making in clinical practice. The sample size was reduced for data on tamoxifen uptake, but sensitivity analyses did not indicate bias. Although the low level of uptake is comparable with other studies,^4^ it might have reduced statistical power. All women were given 3 months to decide whether they would like to initiate tamoxifen, however, some women might not have made their decision at the time of follow-up. These data are self-reported, and therefore uptake estimates might be biased. A number of sociodemographic, clinical, and psychological factors have been reported to be associated with uptake.^4^ We did not explore the quality of clinician–patient communication, which might influence women’s knowledge, understanding, and beliefs about tamoxifen. However, our findings point to potentially modifiable targets to help women make an informed choice regarding preventive therapy.

## Conclusion

In this multicenter study, the decision to initiate tamoxifen was predicted by women’s beliefs about tamoxifen and medicines in general, as well as perceived sensitivity to its negative effects. Eliciting and addressing women’s beliefs might help support informed decision-making.

### Clinical Practice Points

• The effectiveness of preventive therapy for breast cancer depends on adequate uptake, but initiation rates remain low. Across many disease contexts, individuals’ beliefs about medication have been shown to influence treatment decision-making. Little is known about the psychological factors influencing the decision to use chemoprevention.
• Our multicenter prospective study showed that uptake of breast cancer preventive therapy was low. Using LPA, we identified an important subgroup of women who reported low need for preventive therapy and strong medication concerns. These women were less likely to initiate tamoxifen.
• This study identified why some women might decide to opt out of taking tamoxifen as a preventive measure. Identifying and addressing medication beliefs might help support informed decision-making.

## Disclosure

R.J.T. has received honorarium from Novartis. S.G.S. has received consulting fees from Luto Research. The remaining authors have stated that they have no conflicts of interest.

## Figures and Tables

**Supplemental Table 1:** Univariable Comparison of Retention According to Medication Beliefs (n = 408)

Mean (SD)	Baseline Only (n = 150)	Baseline and 3 Months (n = 258)	*P*
BMQ Specific Necessity	2.66 (0.72)	2.61 (0.80)	.549
BMQ Specific Concerns	3.07 (0.61)	3.14 (0.59)	.297
BMQ General Overuse	2.71 (0.72)	2.67 (0.73)	.611
BMQ General Harmfulness	2.30 (0.58)	2.27 (0.63)	.629
PSM	2.32 (0.80)	2.34 (0.75)	.798

*P* value tests for significant differences between baseline and baseline and 3-month cohorts using *t* tests.

Abbreviations: BMQ = Beliefs about Medicines Questionnaire; PSM = Perceived Sensitivity to Medicines Scale.

**Supplemental Table 2:** Correlations Between Medication Belief Variables (n = 408)

	1	2	3	4
Specific Necessity	–	–	–	–
Specific Concerns	−.040	–	–	–
General Overuse	−.099^a^	.293^b^	–	–
General Harmfulness	−.060	.294^b^	.623^b^	–
Perceived Sensitivity to Medicines	−.032	.252^b^	.193^b^	.174^b^

Data presented are Pearson correlation coefficients.

a Correlation is significant at the .05 level.

b Correlation is significant at the .01 level.

**Supplemental Table 3:** Model Fit Statistics for BMQ and PSM Variables (n = 406)

	Class				
	1	2	3	4	5
Parameters	10	16	22	28	34
LL	−2090.764	−1997.354	−1964.527	−1942.876	−1931.403
AIC	4201.528	4026.709	3973.054	3941.752	3930.807
BIC	4241.592	4090.810	4061.194	4053.930	4067.023
Entropy	–	0.666	0.759	0.777	0.730
Sample Size per Class, % (n)	–	Class 1 = 62 (252) Class 2 = 38 (154)	Class 1 = 49.3 (200) Class 2 = 3.4 (14) Class 3 = 47.3 (192)	Class 1 = 9.9 (40) Class 2 = 33 (134) Class 3 = 2.7 (11) Class 4 = 54.4 (221)	Class 1 = 17 (69) Class 2 = 3.0 (12) Class 3 = 32.2 (131) Class 4 = 43.1 (175) Class 5 = 4.7 (19)
VLMR-LRT *P* Value	–	.0001	.2951	.1590	.7835
LMR-LRT *P* Value	–	.0001	.3023	.1652	.7856

Two participants had missing data for all 5 variables and were excluded from the analysis.

Abbreviations: AIC = Akaike Information Criterion; BIC = Bayesian Information Criterion; BMQ = Beliefs about Medicines Questionnaire; LL = log-likelihood; LMR-LRT = Lo–Mendell–Rubin adjusted likelihood ratio test; PSM = Perceived Sensitivity to Medicines Scale; VLMR-LRT = Vuong–Lo–Mendell–Rubin likelihood ratio test.

**Supplemental Table 4:** Model Fit Statistics for BMQ Variables Only (n = 406)

	Class				
	1	2	3	4	5
Parameters	8	13	18	23	28
LL	−1632.263	−1546.829	−1520.696	−1498.641	−1492.457
AIC	3280.526	3119.659	3077.391	3043.283	3040.914
BIC	3312.576	3171.741	3149.505	3135.429	3153.092
Entropy	–	0.661	0.706	0.815	0.790
Sample Size per Class, % (n)	–	Class 1 = 61.3 (249) Class 2 = 38.7 (157)	Class 1 = 12 (49) Class 2 = 62.6 (254) Class 3 = 25.4 (103)	Class 1 = 60.1 (244) Class 2 = 11.1 (45) Class 3 = 1 (4) Class 4 = 27.8 (113)	Class 1 = 26.4 (107) Class 2 = 11.1 (45) Class 3 = 53 (215) Class 4 = 9 (37) Class 5 = 0.5 (2)
VLMR-LRT *P* Value	–	.0000	.1910	.0638	.4124
LMR-LRT *P* Value	–	.0001	.1990	.0673	.4194

Two participants had missing data for all 4 variables and were excluded from the analysis.

Abbreviations: AIC = Akaike Information Criterion; BIC = Bayesian Information Criterion; BMQ = Beliefs about Medicines Questionnaire; LL = log-likelihood; LMR-LRT = Lo–Mendell–Rubin adjusted likelihood ratio test; VLMR-LRT = Vuong–Lo–Mendell–Rubin likelihood ratio test.

**Supplemental Table 5:** Model Fit Statistics for BMQ and PSM Variables, for Baseline and 3 Months (n = 258)

	Class				
	1	2	3	4	5
Parameters	10	16	22	28	34
LL	−1343.119	−1281.764	−1257.355	−1232.145	−1222.712
AIC	2706.238	2595.527	2558.710	2520.291	2513.423
BIC	2741.767	2652.374	2636.875	2619.774	2634.224
Entropy	–	0.719	0.758	0.852	0.862
Sample size per class (%; n)	–	Class 1 = 70 (180) Class 2 = 30 (78)	Class 1 = 50 (129) Class 2 = 46.1 (119) Class 3 = 3.9 (10)	Class 1 = 11.6 (30) Class 2 = 26 (67) Class 3 = 60.9 (157) Class 4 = 1.5 (4)	Class 1 = 11.6 (30) Class 2 = 1.6 (4) Class 3 = 58.5 (151) Class 4 = 26.4 (68) Class 5 = 1.9 (5)
VLMR-LRT *P* Value	–	.0019	.2136	.1444	.7560
LMR-LRT *P* Value	–	.0022	.2215	.1500	.7598

Two participants had missing data for all 4 variables and were excluded from the analysis.

Abbreviations: AIC = Akaike Information Criterion; BIC = Bayesian Information Criterion; BMQ = Beliefs about Medicines Questionnaire; LL = log-likelihood; LMR-LRT = Lo–Mendell–Rubin adjusted likelihood ratio test; PSM = Perceived Sensitivity to Medicines Scale; VLMR-LRT = Vuong–Lo–Mendell–Rubin likelihood ratio test.

**Supplemental Table 6:** Medication Belief Group Membership According to Participant Characteristics and Univariable and Multivariable Logistic Regression Model (n = 406)

	Group 2: Low Need, Higher Concerns, n (%)	Univariable		Multivariable	
		OR (95% CI)	*P*	OR (95% CI)	*P*
**Age**					
≤35 years	16 (39)	0.70 (0.34-1.46)	.346	0.65 (0.28-1.56)	.337
36-49 years	87 (33.7)	0.56 (0.35-0.88)	**.013**	0.56 (0.34-0.93)	**.024**
≥50 years	51 (47.7)	Ref		Ref	
**Children**					
Yes	115 (36.6)	0.79 (0.49-1.26)	.317	0.88 (0.49-1.57)	.653
No	39 (42.4)	Ref		Ref	
**Ethnic Group**					
White	140 (36.5)	Ref		Ref	
Other	11 (61.1)	2.74 (1.04-7.23)	**.042**	2.40 (0.81-7.14)	.117
**Education Level**					
Degree or above	58 (33)	0.74 (0.49-1.11)	.143	0.71 (0.44-1.13)	.148
Below degree level	89 (40.1)	Ref		Ref	
**Health Status**					
Poor	8 (50)	1.64 (0.55-4.92)	.378	1.50 (0.43-5.25)	.526
Fair	38 (48.7)	1.56 (0.80-3.04)	.192	1.32 (0.64-2.72)	.457
Good	80 (33.3)	0.82 (0.47-1.44)	.491	0.69 (0.38-1.27)	.234
Excellent	25 (37.9)	Ref		Ref	
**Risk Level**					
Moderate	96 (39.7)	1.20 (0.79-1.81)	.395	1.41 (0.89-2.23)	.144
High	56 (35.4)	Ref		Ref	
Unclear^a^	2 (33.3)	–		–	
**SES**					
Low (most deprived)	56 (47.1)	1.63 (0.99-2.66)	.052	1.20(0.69-2.08)	.525
Middle	43 (32.8)	0.89 (0.55-1.47)	.658	0.77 (0.45-1.33)	.352
High (least deprived)	53 (35.3)	Ref		Ref	
**Employment**					
Full-time	125 (35.9)	Ref		Ref	
All other employment	29 (50)	1.78 (1.02-3.12)	**.043**	1.39 (0.74-2.62)	.313
**Marital Status**					
Married or cohabiting	100 (33.6)	Ref		Ref	
Unmarried	51 (49.5)	1.94 (1.23-3.06)	**.004**	1.63(0.96-2.76)	.071

Bold *P* values indicate statistical significance *P* < .05.

Abbreviation: OR = odds ratio; Ref = reference; SES = socioeconomic status.

a Category not included in univariable and multivariable analyses because of insufficient cases. The multivariable model included 379 respondents.

**Supplemental Table 7:** Uptake of Tamoxifen According to Clinic Setting (n = 258)

Clinic Setting	Uptake of Tamoxifen, % (n)
Genetics	6.7 (1/15)
Breast Clinic	6.9 (2/29)
Family History	15.5 (28/181)
Family History Clinic and Genetics	21.2 (7/33)

## Supplemental Data

Data Profile
